# What goes up must come down: Using weather surveillance radar data to forecast migratory birds banded on the ground

**DOI:** 10.1002/ecy.70565

**Published:** 2026-11-05

**Authors:** Carolyn S. Burt, Sara R. Morris, Siddharth Chidambaram, Kyle G. Horton

**Affiliations:** ^1^ Department of Forestry and Natural Resources Purdue University West Lafayette Indiana USA; ^2^ UNH School of Marine Sciences and Ocean Engineering, Morse Hall Durham New Hampshire USA; ^3^ Appledore Island Migration Station and Shoals Marine Laboratory (Joint Program of University of New Hampshire and Cornell University) Durham New Hampshire USA; ^4^ Grainger School of Engineering University of Illinois Urbana‐Champaign Champaign Illinois USA

**Keywords:** aeroecology, avian migration, bird banding, ecological forecasting, stopover, weather surveillance radar

## Abstract

Ecological forecasting is a powerful framework to predict future states of ecological systems, including species distributions and supporting proactive conservation, management, and policy decisions that exist in a rapidly changing world. Forecasting efforts span a diversity of systems and organisms, yet many ecological phenomena, especially those involving highly mobile species, remain difficult to predict. Migration, for example, shapes population dynamics, species interactions, ecological services, and disease spread across terrestrial and aerial environments. Migratory pulses of birds observed in airspaces and stopover habitats are often studied separately; however, the connection between these two phases of migration remains largely unquantified. Here, we evaluate whether weather surveillance radar (WSR) measures of nocturnal migration activity can predict the number of birds captured at a long‐term bird banding station, thereby bridging aerial and terrestrial perspectives of migration. Using data from two WSR sites—KBOX (Boston, MA) and KGYX (Portland, ME)—and bird banding records from Appledore Island, Maine (1996–2023), we examined how radar‐derived measures of aerial migration activity forecast terrestrial bird banding captures. Using leave‐one‐year‐out cross‐validation, generalized additive models incorporating radar measures, atmospheric conditions, and non‐environmental covariates produced skillful probabilistic forecasts of daily captures in both seasons, improving on a persistence baseline in 21 of 23 spring and 23 of 26 fall years (median CRPS skill 0.25 and 0.12). Including radar‐derived measures further improved forecasts beyond atmospheric predictors in the majority of years (spring: 18 of 23; fall: 21 of 26). The models explained 57.8% and 49.9% of the deviance in daily spring and fall captures, respectively. These findings show that linking aerial and terrestrial monitoring can enhance migration forecasts, with implications for conservation strategies such as targeted “lights out” initiatives and stopover habitat management. This approach offers a scalable pathway to forecasting the connection between large‐scale atmospheric data and ground‐level ecological processes.

## INTRODUCTION

The movement of organisms is a fundamental ecological process that redistributes energy, competition, nutrients, species interactions, and disease across the planet (Alerstam & Bäckman, 2018; Nathan et al., 2008; Somveille et al., 2018). These movements, ranging from the daily foraging of individuals to continent‐spanning migrations, shape ecosystems and have cascading impacts on biodiversity, ecosystem services, and human well‐being. Seasonal migrations, while broadly predictable over large spatial and temporal scales, often exhibit striking variability at finer resolutions (Jimenez et al., 2025). Near‐term ecological forecasting provides a critical framework to anticipate these movements and the ecological processes that drive them, enabling both advances in basic ecological understanding and actionable conservation strategies (Dietze et al., 2018).

Bird migration provides a natural application for ecological forecasting, given its dynamic nature across space and time and its coupling of aerial and terrestrial habitats. Most migratory species cannot fly between breeding and nonbreeding grounds in a single, sustained flight (Newton, 2008). Instead, their journeys through vast airspaces are punctuated by critical stopovers to rest, refuel, and recover in essential terrestrial habitats (Alerstam, 2003; Bonter et al., 2009; Cohen et al., 2021; Hedenström & Alerstam, 1997). Migration activity, whether observed in flight or on the ground, often exhibits striking temporal variation, characterized by saltatory pulses of movement during spring and fall (Horton, Van Doren, Stepanian, Farnsworth, & Kelly, 2016). While aerial and terrestrial phases of migration are typically studied in isolation (Buler & Dawson, 2014; Dokter et al., 2018; Horton et al., 2020; Jonzén et al., 2006), understanding the connections between them is vital to unraveling the complexity of avian migration. For instance, does a pulse of activity in one habitat predict a lagged response in another? This question highlights the need to integrate observations across landscapes to fully comprehend migration dynamics.

In many parts of the world, capture techniques such as mist netting have long been foundational for studying bird migration across terrestrial landscapes. Systematic, seasonal bird banding programs—some extending over half a century—have yielded invaluable insights into migration phenology (Horton, Morris, et al., 2023; Neate‐Clegg & Tingley, 2023), migratory connectivity (Gregory et al., 2023), and bird community dynamics during migration (DeSimone et al., 2024). Juxtaposed with the volume of individuals captured at these long‐term banding stations, tracking studies of individual birds using miniature tags (e.g., GPS) provide a wealth of complementary information, at times at global scales (Jetz et al., 2022). However, the intensive effort required for such approaches presents challenges for scaling to the continental level, which is necessary to fully understand the spatial and temporal dynamics of migration. In contrast, participatory science data, such as observations collected through eBird, provide broad‐scale insights across species, enabling the study of population declines (Fink et al., 2023) and shifts in phenology (Youngflesh et al., 2021) in response to environmental change at continental scales (Sullivan et al., 2014). Despite its extensive coverage, this semi‐structured approach can miss finer details of migration dynamics. For example, irregular sampling efforts may fail to capture the nuanced ebb and flow of daily migratory movements throughout a season, especially when individuals are unmarked, as is the case with eBird observations. Further, information about the age, sex, and condition of birds is lacking in most observations. This highlights that while participatory science maximizes scale, and mist netting offers depth and precision—no single approach, even in terrestrial habitats, is a perfect solution to studying seasonal migration.

Meanwhile, studying migration through airspaces comes with its own challenges. Weather surveillance radar (WSR) has been used for decades to measure and predict where migratory birds will be in the airspace during nocturnal spring and fall migrations (Gauthreaux, 1971; Horton, Van Doren, Stepanian, Farnsworth, & Kelly, 2016; Kelly & Horton, 2016; Lippert et al., 2022; Nilsson et al., 2019). WSR can be used to study discrete stopover events captured by nightly departures from terrestrial habitats (Bonter et al., 2009; Buler & Dawson, 2014; Cohen et al., 2021) or the broad front movements through airspaces that occur throughout the night (Giuntini et al., 2025). Recently, using WSR data, Nussbaumer et al. (2024) were able to connect nightly migratory movements with diurnal stopover abundance by quantifying birds landing and departing on the landscape (Nussbaumer et al., 2024). Moreover, through this novel flow model approach, they were able to estimate that migratory turnover averaged about 13%, but was as high as 50% on peak nightly movements. However, a key limitation of WSR is its general inability to resolve species‐specific information. Exceptions include specific behaviors, such as roosting by aerial insectivores (e.g., martins and swallows) (Belotti et al., 2023; Bridge et al., 2015; Deng et al., 2023; Kelly et al., 2012), known species‐specific exoduses like Eared Grebe during winter migrations in Utah, USA (Williams & Laird, 2018), and waterfowl movements (O'Neal et al., 2010). To address this limitation, additional methodologies, such as recorded flight calls, can better identify species‐level comparisons, as well as variations in calls across individuals, and have been used to either integrate or validate WSR data (Horton et al., 2015; Lostanlen et al., 2024; Osterhaus et al., 2025; Ress et al., 2024). Together, both methods—radar and flight call monitoring—are widely used tools available for monitoring nocturnally migrating birds in the airspace, with each method contributing unique strengths to our understanding of migration dynamics.

Understanding how terrestrial and aerial habitats are linked is critical for migratory bird conservation efforts. Across the continental United States, ecological forecasts are generated multiple times per day to predict migration activity (Horton et al., 2021; Van Doren & Horton, 2018), and can be used to guide lights out initiatives to mitigate terrestrial‐based collisions with built infrastructure or motivate ground‐based observations (Burt et al., 2023). However, even with improved forecast models that integrate terrestrial habitat and structure (Jimenez et al., 2025, 2026), these models primarily predict when birds will be present in the airspace at night, rather than their distribution on the ground the following morning. Previous studies have examined relationships between nocturnal migration intensity and next‐day stopover density (e.g., Fischer et al., 2012; Horton, Shriver, & Buler, 2016), but the strength of these relationships have been mixed, and these studies did not explicitly evaluate the potential for forecasting migratory movements. As a result, a predictive connection between aerial migration dynamics and subsequent terrestrial outcomes has remained limited for applied conservation. Being able to predict birds on the ground using radar data would be the first time these two habitats (aerial and terrestrial) have been linked in this direct way through ecological forecasting.

To this end, we evaluated whether WSR measures of nightly migration activity can forecast the number of birds captured at a long‐term bird banding station, thereby linking aerial and terrestrial systems. To assess this, we used data from two WSR sites—KBOX (Boston, Massachusetts) and KGYX (Portland, Maine)—alongside bird banding records from Appledore Island, Maine, spanning 1996–2023. We predicted a positive association between radar‐based measures of migration intensity and on‐the‐ground mist‐net capture rates, hypothesizing that aerial activity on the preceding night (migration intensity, speed, and direction) would be predictive of terrestrial activity. Recognizing the critical role of abiotic factors in shaping migration dynamics (Kemp et al., 2013; Liechti, 2006; Richardson, 1978; Van Doren & Horton, 2018), particularly along coastal sites (Horton, Van Doren, Stepanian, Hochachka, et al., 2016), we incorporated wind speed, wind direction, temperature, and visibility as additional predictors of spring and fall capture rates. Using this suite of aerial predictors—including weather variables and WSR data—we aimed to forecast aggregate bird captures of all species and test the capacity to predict species‐specific patterns of birds banded in both seasons at Appledore Island. By integrating radar and banding data, this study bridges the gap between aerial and terrestrial perspectives of migration.

## MATERIALS AND METHODS

### Bird capture and banding

We focused our analysis on songbird migration on Appledore Island, Maine (42.990100° N, 70.615137° W, Figure 1), a 38‐hectare island located 10 km off the coast of Maine and the largest in the Isles of Shoals group. Approximately two‐thirds of the island consists of coastal scrub shrub habitats and low trees, while the remaining third is exposed rock (Horton & Morris, 2012). While the Appledore Island Migration Station operated well before 1996, we restricted our analyses from 1996 to 2023 to align with existing WSR data from nearby KBOX and KGYX (see next section). Banding was not conducted in spring and fall of 2020 because of the COVID‐19 pandemic. Appledore Island Migration Station operated primarily from early May to mid‐June (on average May 10th to June 8th, 29 days) during spring migration and from mid‐August to late September (on average August 17th to September 20th, 34 days) during fall migration. Typically, 5–10 mist nets (12 × 2.6 m, 30‐mm mesh) were used daily to capture birds. Nets were opened just prior to sunrise and closed shortly after sunset and checked at least every 30 min throughout the day. Nets were occasionally closed during inclement weather, such as rain, strong winds, or heavy fog. We recorded the opening and closing of each mist net to the nearest 5 min, and we calculated net hours by summing the number of hours we operated each net each day (Morris et al., 1994). We removed banding days that had less than 50 net hours (median net hours: spring 122 h, fall 124 h).

**FIGURE 1:**
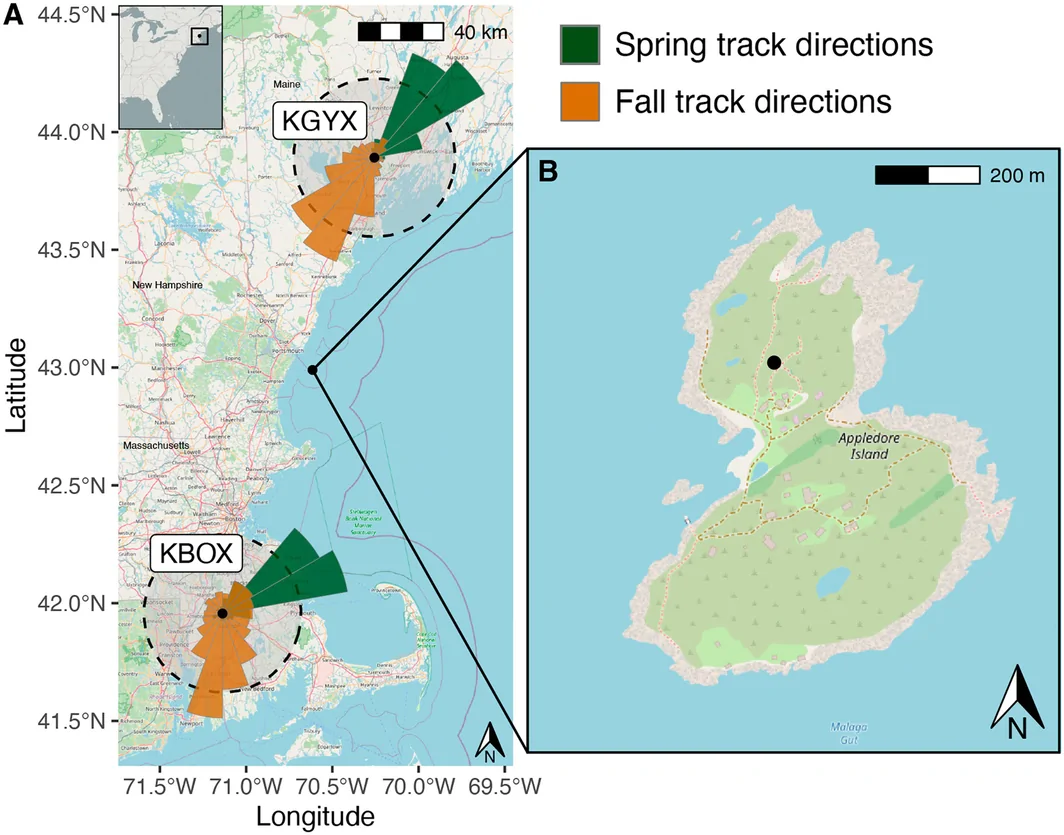
(A) Overview of study region, showing two NEXRAD sites (KBOX, Boston, MA and KGYX, Portland, ME) and bird banding site, Appledore Island, ME. (B) Zoom‐in of bird banding site, with black dot approximately the location of mistnets. Circular buffers show 37.5‐km buffer used for radar extractions of radar reflectivity (migration intensity) and radial velocity (track direction and ground speed). Rose diagrams depict seasonal distributions of migrant track from 1996 to 2023 at 20° intervals and weighted by migration traffic rate (reflectivity × groundspeed).

All captured birds were fitted with a U.S.G.S. band, and we recorded mass, wing chord, tarsus, fat score, and age and sex when possible. For our analysis, we only used newly banded birds to calculate the daily number of birds banded. While some species are known to breed on the island (e.g., Common Yellowthroat, *Geothlypis trichas*; Song Sparrow, *Melospiza melodia*), we did not exclude these species because the volume of new captures (of these species) strongly suggests passage migrants.

### Weather surveillance radar

We used measures from WSR north (KGYX) and south (KBOX) of Appledore Island Migration Station. We downloaded Level‐II radar data from the Amazon Web Services online repository (https://unidata-nexrad-level2.s3.amazonaws.com/index.html). We downloaded radar scans 3.5 h after local sunset, a time when radar reflectivity at these two sites has been shown to peak (Giuntini et al., 2025). Using the bioRad package in R (Dokter et al., 2019), we built vertical profiles of radar reflectivity, ground speed, and flight direction from 5 to 37.5 km away from the radar using the five lowest elevation scans. We integrated these vertical profiles using the *integrate_profile* function and extracted vertically integrated reflectivity and the zonal (u) and meridional (v) components of ground speed from radial velocity as predictors of the number of birds banded the following day.

### Weather covariates

We extracted atmospheric conditions from the North American Regional Reanalysis (NARR) dataset (Mesinger et al., 2006). NARR provides a broad diversity of modeled atmospheric measures across a 32‐km grid that is assimilated at 3‐h intervals. We selected wind speed and direction (zonal and meridional), air temperature, and visibility as predictors of the number of birds banded. For wind speed and temperature measures, we selected data from 900‐hPa (mean 1019 m a.g.l.), 950‐hPa (mean 564 m a.g.l.), and 1000‐hPa (mean 128 m a.g.l) pressure levels. We selected a single 32‐km grid cell most closely centered on the Appledore Island Migration Station and used measures from 9 UTC, the time just shortly before banding started in the morning.

### Forecast model

We constructed generalized additive models (GAMs) in R using the mgcv package to predict daily numbers of birds banded in spring and fall (Wood, 2015). Because daily counts were over dispersed, we used a negative binomial error distribution with a log link. We used GAMs because we predicted the relationships between predictors and daily banding counts to be nonlinear and smooth, rather than strictly linear. GAMs provided a flexible, nonparametric framework for modeling such relationships. As predictors, we included radar measures of the log of vertically integrated reflectivity and u and v components of migrant ground speed from both KBOX and KGYX. We log‐transformed vertically integrated reflectivity because the distribution was heavily right skewed. We included air temperature, zonal (u, west to east) and meridional (v, south to north) components of wind, visibility, ordinal date, net hours, and number of birds banded the previous day as additional predictors. We modeled each predictor with a penalized regression spline (basis dimension *k* = 3), fitting the models by restricted maximum likelihood (REML) and applying an additional penalty on each smooth's null space (select = TRUE) so that uninformative terms could be shrunk toward zero (Wood, 2011). Because pressure level predictors were highly correlated (*r* > 0.5, e.g., u‐wind 950 hPa~u‐wind 1000 hPa), we examined full model performance using one of the three pressure levels (900, 950 or 1000 hPa) for zonal wind, meridional wind, and air temperature. We selected the pressure level that resulted in the greatest deviance explained. For both spring and fall, we selected 900‐hPa variables. Rather than selecting a reduced model, we retained all predictors and relied on the penalized fit (select = TRUE) to shrink uninformative terms, prioritizing out‐of‐sample predictive performance over parsimony. Lastly, we only included a season if at least 10 days of banding, radar, and atmospheric observations were available. In total, we included 23 spring seasons and 26 fall seasons.

To validate our final models, we used leave‐one‐year‐out cross‐validation: We set aside all data from a single year for evaluation, trained the GAM on the remaining years, and repeated this for every year in the record. For each held‐out year, we generated independent daily predictions of the number of birds banded, assuming 123 net hours (approximately the median effort in both spring and fall). We applied the same procedure to the 10 most banded species per season. Forecast performance was evaluated primarily with the continuous ranked probability score (CRPS, see following section), but root mean squared error (RMSE) and Pearson's correlation were also used as intuitive secondary summaries.

We used leave‐one‐year‐out cross‐validation to maximize the amount of training data and holdout trials as opposed to an end‐sample holdout validation as an end‐sample approach would restrict our number of trials and has variable training lengths of data. We further note that our models contain no year term and therefore cannot exploit long‐term trends, limiting the scope for information leakage between folds. To confirm that our conclusions are not an artifact of the leave‐one‐year‐out scheme, which can train on years later than the one being predicted, we additionally evaluated the models under an expanding‐window (end‐sample holdout) forecast, in which each year was predicted using only the years preceding it, a true out‐of‐sample forecast (See Appendix S1). Under this scheme the full model retained positive CRPS skill relative to the baseline in nearly all forecast years, confirming that our conclusions are robust to the validation approach.

To visualize the modeled relationships between each predictor and the number of birds banded, we constructed partial dependence plots for all 13 predictors. For each plot, we varied the focal predictor across its observed range (5th–95th percentiles) while holding the remaining predictors at their median values (net hours fixed at 123) and generated predictions on the response scale. To rank predictor importance, we used a leave‐one‐term‐out approach: We refit the model with each predictor removed in turn and computed the change in Akaike information criterion (AIC) relative to the full model (ΔAIC = AIC_reduced − AIC_full), where larger values indicate greater importance (Burnham & Anderson, 2002).

### Skill scores for model improvement

To evaluate whether atmospheric and radar predictors improve forecasts of daily banding totals, we compared three nested models per season. The baseline model contained only structural covariates (non‐environmental) which included previous‐day captures, sampling effort (net hours), and seasonal timing (ordinal date). The atmospheric model added lower atmosphere weather conditions (visibility, air temperature, and u‐ and v‐wind components), and the full model additionally included radar‐derived measures (reflectivity and u‐ and v‐flight components from both radar sites). Models were fit and evaluated using leave‐one‐year‐out cross‐validation.

We evaluated forecasts with two complementary metrics, each expressed as a skill score relative to the baseline. Point‐accuracy summaries such as the correlation coefficient and root mean square error describe how close a single predicted value is to the observation, but they ignore a central feature of a forecast—its uncertainty (Gneiting et al., 2007; Gneiting & Raftery, 2007; Simonis et al., 2021). To capture this, we used the CRPS, which evaluates the entire predictive distribution rather than a single point, alongside RMSE as an intuitive point‐accuracy complement.

RMSE is the square root of the mean squared difference between observed counts and the expected value (mean) of each forecast's predictive distribution, in units of daily captures. The CRPS (Gneiting & Raftery, 2007; Matheson & Winkler, 1976) generalizes the mean absolute error to probabilistic forecasts: It measures the integrated squared difference between a forecast's predictive cumulative distribution function, *F*, and a step function placed at the observed value, *y*.

$$\text{CRPS}\left(F,y\right)=\int_{-\infty }^{\infty }{\left[F\left(x\right)-1\left(x\geq y\right)\right]}^{2}\mathrm{dx}$$

where 𝟙(*x* ≥ *y*) is the indicator (step) function that jumps from 0 to 1 at the observation. CRPS is expressed in the same units as the response (daily captures) and, for a deterministic point forecast, reduces exactly to the absolute error. It is a proper scoring rule that is optimized only by the forecast's true predictive distribution, so it cannot be improved by over‐ or under‐stating uncertainty. CRPS jointly rewards accuracy, calibration, and sharpness, with lower values indicating better forecasts. For either metric (CRPS skills and RMSE skill), we expressed forecast skill relative to the baseline as:

$$\text{CRPS skill}=1-\frac{{\text{CRPS}}_{\text{model}}}{{\text{CRPS}}_{\text{baseline}}}$$

where positive values indicate improvement over the baseline and zero indicates equivalent performance. To compute CRPS, we generated each forecast's predictive distribution by drawing from the joint posterior distribution of the model coefficients and propagating this uncertainty through the negative binomial observation model. CRPS was then calculated from the resulting posterior‐predictive samples using the crps_sample function in the scoringRules package (Jordan et al., 2019). For analyses requiring many repeated model fits, such as the training‐length experiment (see next section) and the species‐level forecasts, we computed CRPS analytically from each forecast's negative binomial predictive distribution using the fitted mean and estimated dispersion, rather than by posterior simulation. This approach is computationally efficient but conditions on the fitted mean and therefore does not propagate parameter uncertainty.

### Forecast sensitivity to number of training years

To assess how forecast performance depends on the length of the training record, we conducted a training‐set subsampling experiment for each season. We repeated the following procedure 50 times: In each replicate, we randomly set aside 3 years as a held‐out evaluation set and trained the full model on random subsets of the remaining years, ranging from 2 to 20 years in length (k), scoring each model's forecasts on the same three held‐out years. Within a replicate, the same 3 years are predicted at every training‐set size, so differences in performance reflect the amount of training data rather than which years were withheld; across replicates, the withheld years differ, so the result does not depend on any single choice of evaluation years. Performance for each replicate was summarized with the CRPS, computed from the negative binomial predictive distribution of each daily forecast. To identify the point beyond which additional years no longer improved forecasts, we fit a segmented (piecewise‐linear) regression of CRPS on training‐set size using the *segmented* package (Muggeo, 2003), which estimates a breakpoint separating an initial phase of steep improvement from a subsequent plateau. Because the analytic CI for a breakpoint can be unreliable when the slope change is gradual, we quantified its uncertainty with a nonparametric bootstrap: We resampled the replicate‐level CRPS values with replacement (500 iterations), refit the segmented model to each resample, and took the 2.5th and 97.5th percentiles of the breakpoints as a 95% CI. Single‐year training sets were excluded from the breakpoint fit, as 1 year is insufficient to constrain the model and produced unstable predictions.

## RESULTS

After merging banding data from 1996 to 2023 with available radar observations, and assuring banding occurred the previous day, we included 63,963 birds of 144 species banded at the Appledore Island Migration Station, 39,237 during spring, and 24,726 during fall. In total, we included 481 spring sampling days in our analysis and 689 in the fall. During spring, 81.6 ± 68.6 (±SD) birds were banded per day on average, ranging from 1 to 400 and 35.9 ± 25.3 (±SD) birds banded per day in fall, ranging from 1 to 185. The weighted mean of spring track direction was 59.8° for KBOX and 42.3° for KGYX, and 173.7° for KBOX and 221.2° for KGYX during the fall (Figure 1).

### Skill scores

Across leave‐one‐year‐out cross‐validation, the full model incorporating radar measurements consistently improved forecast skill relative to a structural baseline in both migration seasons (Figure 2). Models including atmospheric predictors alone also improved performance relative to the baseline, but the full model more frequently achieved higher skill than the atmospheric model, indicating additional predictive information from radar‐derived variables.

**FIGURE 2:**
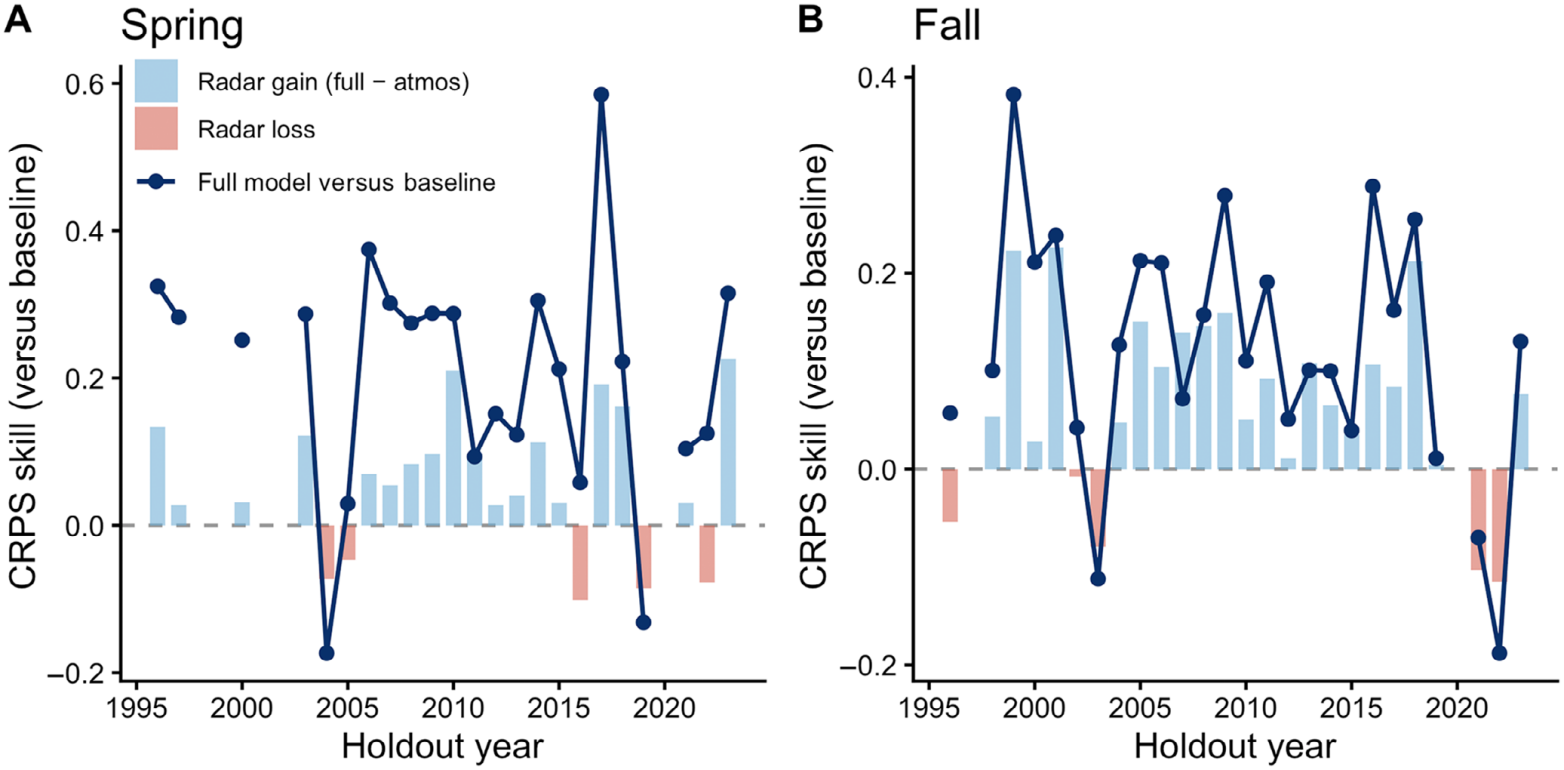
Year‐by‐year forecast skill and the contribution of weather surveillance radar, by season. Models were evaluated by leave‐one‐year‐out cross‐validation. Lines and points show the CRPS skill of the full (radar‐inclusive) model relative to a persistence baseline, where skill = 1 − (CRPS_model/CRPS_baseline). In both plots, 0 (dashed line) equals baseline performance and higher is better. Bars show the radar‐specific contribution each year (Δ = CRPS skill of the full model minus that of an equivalent model without radar). Blue indicates radar improved the forecast and red indicates it did not. Panels are faceted by season (A, spring; B, fall) and breaks in the line and absent bars denote seasons excluded for insufficient sampling.

During spring migration, probabilistic forecast performance for the full model improved relative to the baseline in 21 of 23 holdout years (positive CRPS skill), with a median CRPS skill of 0.25 (IQR 0.11–0.30) and skill exceeding 0.2 in 14 of 23 years (Figure 2A). Relative to the atmospheric model, the full model achieved higher CRPS skill in 18 of 23 years, with a median improvement of 0.06 (IQR 0.03–0.12), demonstrating that radar predictors improved probabilistic forecasts in the majority of spring seasons. Point prediction accuracy showed a similar pattern, with positive RMSE skill relative to the baseline in 18 of 23 years (median 0.19) and higher RMSE skill than the atmospheric model in 14 of 23 years, although the magnitude of improvement varied among years.

In fall migration, gains relative to the baseline were more modest in magnitude but remained widespread. The full model achieved positive CRPS skill relative to the baseline in 23 of 26 holdout years, with a median CRPS skill of 0.12 (IQR 0.05–0.21), indicating consistent but smaller probabilistic gains than in spring (Figure 2B). Relative to the atmospheric model, CRPS skill was higher for the full model in 21 of 26 years, with a median improvement of 0.07 (IQR 0.02–0.13)—a radar‐specific contribution comparable to that in spring. Improvements in point prediction accuracy were also widespread, with positive RMSE skill relative to the baseline in 23 of 26 years (median 0.11) and higher RMSE skill than the atmospheric model in 21 of 26 years.

Together, these results demonstrate that while atmospheric conditions explain substantial variation in migration‐related banding totals, radar‐derived predictors provide complementary information that improved both probabilistic and point forecasts in most years. Overall forecast skill relative to the baseline was strongest in spring (median CRPS skill 0.25 vs. 0.12 in fall), whereas the radar‐specific improvement over the atmospheric model was comparable across seasons (median CRPS gain 0.06 in spring, 0.07 in fall).

### Spring models

Our final spring GAM, fit to all years, explained 57.8% of the deviance (adjusted *R* ^2^ = 0.47, negative binomial θ = 3.27, *n* = 481 days; full term‐level summary in Appendix S1: Table S1). In our leave‐one‐variable‐out importance assessment, the five most important predictors were ordinal date, zonal (W–E) wind, air temperature, vertically integrated reflectivity at KBOX, and meridional (S–N) wind. Broadly, zonal wind speed, air temperature, KBOX vertically integrated reflectivity, KBOX zonal (W–E) ground speed, and previous‐day captures were positively associated with the number of birds banded (Appendix S1: Figure S1, Table S2). Warmer nights with flight and wind components oriented more easterly predicted more captures, particularly when more birds had been banded the previous day. Migration activity generally peaked mid‐season, around ordinal date 142 (May 23).

Using leave‐one‐year‐out cross‐validation, we generated out‐of‐sample predictions of daily banding totals for each withheld year from radar, atmospheric, and non‐environmental predictors. Pooling predictions across all withheld years, the Pearson's correlation between daily predicted and observed totals was *r* = 0.67 (*p* < 0.001, *n* = 481; Figure 3A). On an annual basis, correlations averaged 0.69 ± 0.10 (SD; range 0.49–0.96) and RMSE averaged 50.6 ± 9.5 (range 28.6–73.5).

**FIGURE 3:**
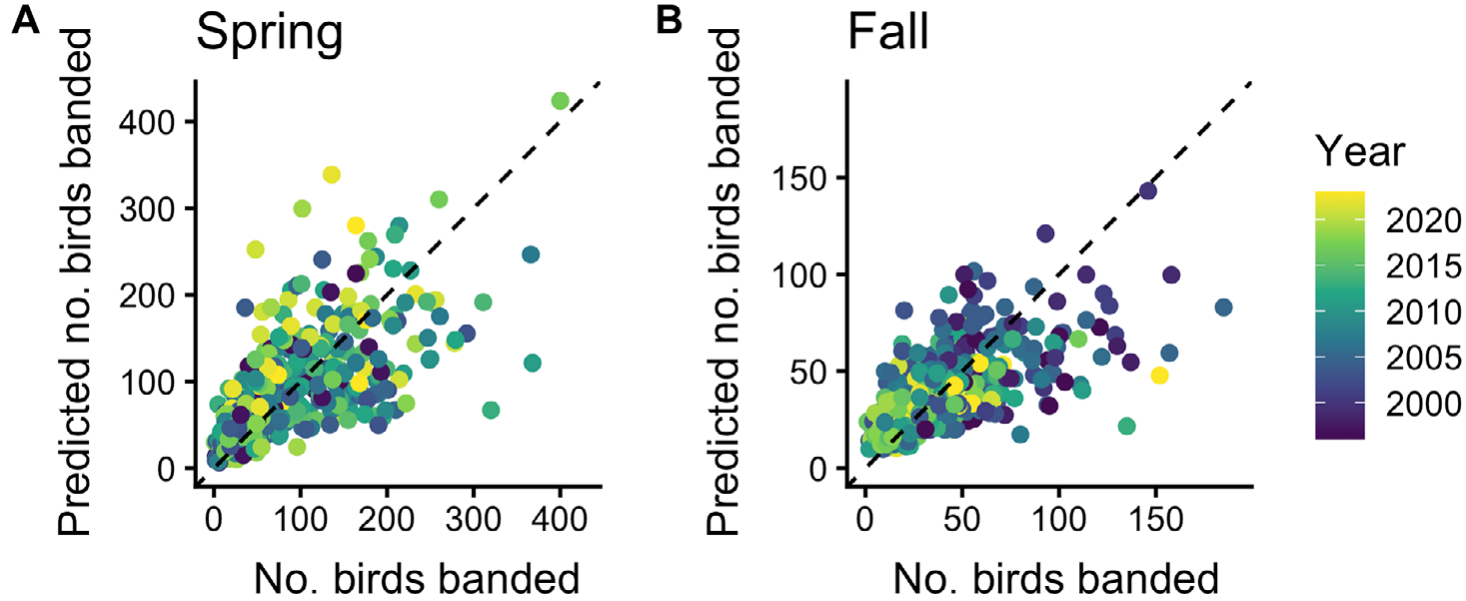
Predicted versus observed daily counts of birds banded on Appledore Island, Maine, during (A) spring and (B) fall migration. Predictions were generated from generalized additive models trained on all years of seasonal data with the focal year withheld, simulating an out‐of‐sample seasonal forecast. Points are colored by year and the dashed line denotes 1:1 agreement between predicted and observed values.

### Fall models

Our final fall GAM, fit to all years, explained 49.9% of the deviance (adjusted *R* ^2^ = 0.44, negative binomial θ = 5.66, *n* = 689 days; full term‐level summary in Appendix S1: Table S3). In our leave‐one‐variable‐out importance assessment, the five most important predictors were previous‐day captures, net hours, meridional (S–N) wind speed, ordinal date, and vertically integrated reflectivity at KGYX (Appendix S1: Figure S2, Table S2). Previous‐day captures had an outsized influence on model performance, with a ΔAIC roughly eight times that of the next most important predictor, net hours. Most top predictors were positively associated with the number of birds banded, except meridional wind speed, for which captures peaked at weak wind speeds. Thus, captures were higher following days with more captures, with greater sampling effort (net hours), and when more migrants were detected aloft by the KGYX and KBOX radars. Captures also increased with ordinal date, suggesting that the seasonal peak in banding likely occurred after the banding season had ended.

Following the same approach as spring modeling, we iteratively held out a year of interest and retrained our fall model for prediction onto the year of interest. Pooling predictions across all withheld years, the Pearson's correlation coefficient between daily predicted number of birds banded and the true number of birds banded was *r* = 0.64 (*p* < 0.001, *n* = 689, Figure 3B). On an annual basis, Pearson's correlations averaged 0.58 ± 0.2 (SD; range 0.11–0.81) and RMSE averaged 18.8 ± 5.9 (range 8.3–32.9).

### Species‐specific models

For spring and fall, we identified the 10 most frequently banded species and built individual, species‐specific models using the same predictors as our aggregate seasonal models. The species set differed between seasons, though five were shared (15 species modeled in total). Forecast performance, summarized as CRPS normalized by each species' mean daily count (lower = better), was broadly comparable across seasons, though marginally stronger in spring (mean normalized CRPS 0.52 vs. 0.57 in fall; Figure 4). The best forecast species were consistent across seasons, with Gray Catbird and Common Yellowthroat ranking among the top performers in both spring (normalized CRPS 0.39 and 0.46) and fall (0.39 and 0.46). In two example time series, predicted Magnolia Warbler daily capture totals tracked observed captures in both timing and magnitude, with observations largely falling within the forecast's predictive interval (Figure 5A: *r* = 0.85, CRPS = 4.8, RMSE = 8.2; Figure 5B: *r* = 0.57, CRPS = 4.1, RMSE = 9.6).

**FIGURE 4:**
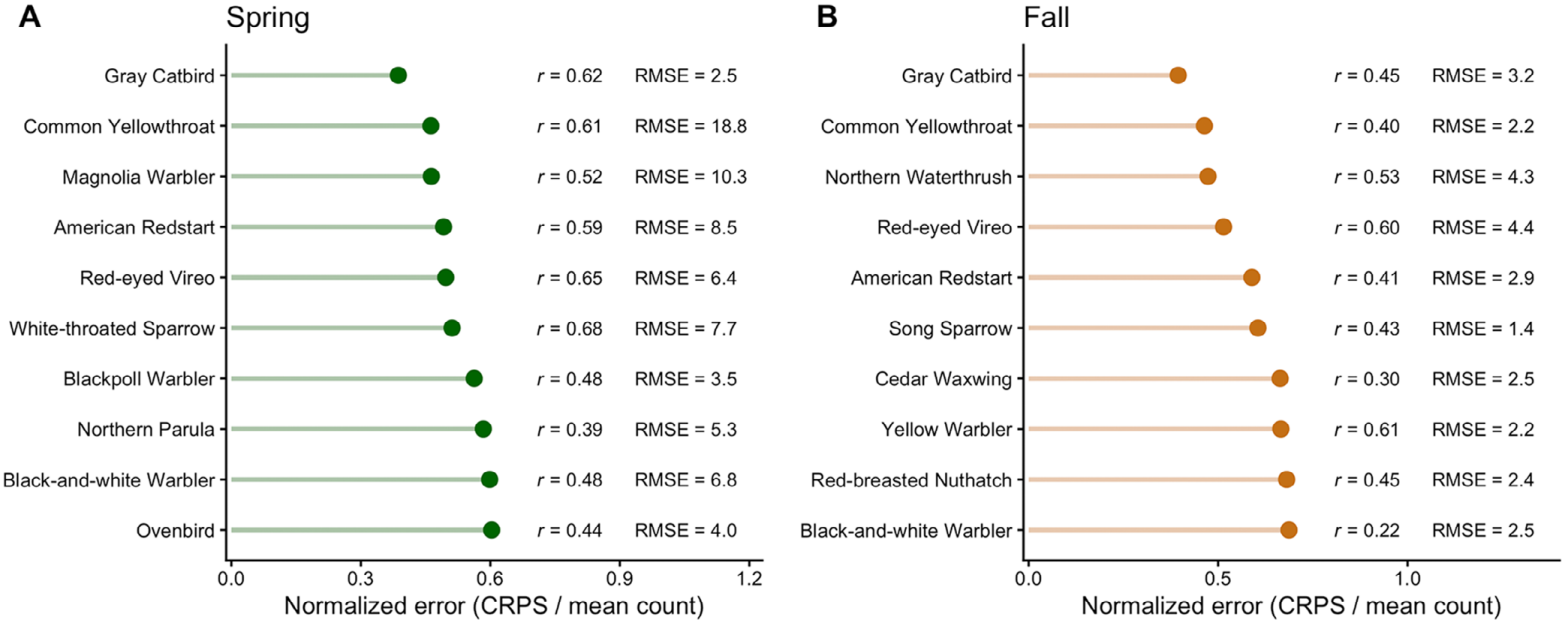
Species‐specific forecast performance for the 10 most frequently banded species in (A) spring and (B) fall. Points show the continuous ranked probability score (CRPS) normalized by each species' mean daily count, a measure of probabilistic forecast error scaled to abundance so that species of differing commonness are comparable (lower = better forecast). Species are ordered from best (lowest normalized CRPS, top) to worst (bottom). Pearson's correlation coefficient (*r*) and root mean square error (RMSE) for each species are given as text. Forecasts were generated using leave‐one‐year‐out cross‐validation.

**FIGURE 5:**
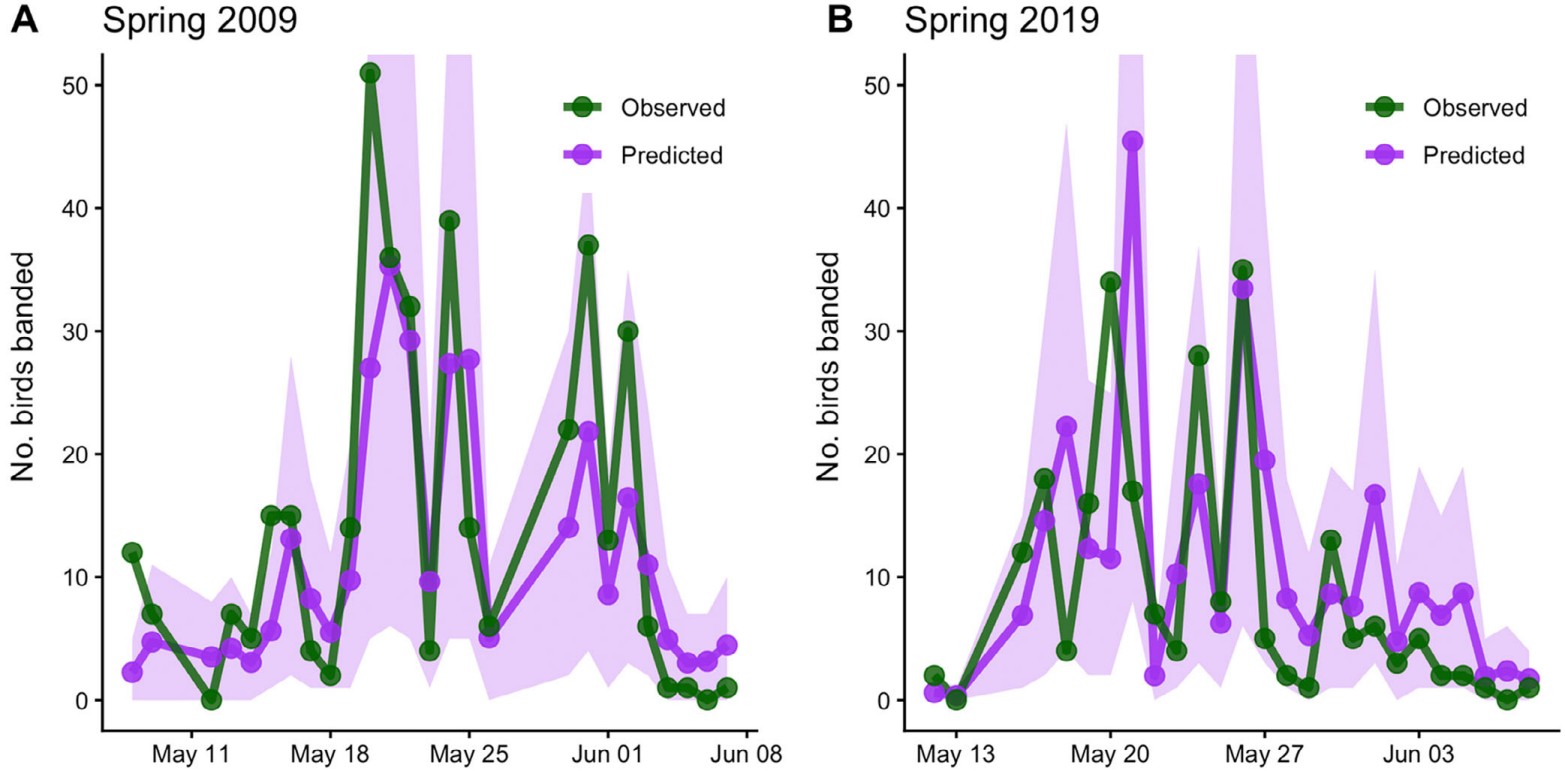
Predicted (purple) and observed (green) daily counts of Magnolia Warblers banded during (A) spring 2009 (*r* = 0.85, CRPS = 4.8, RMSE = 8.2) and (B) spring 2019 (*r* = 0.57, CRPS = 4.1, RMSE = 9.6). In each panel, the model was trained on all other years, with the displayed year held out. The shaded purple band shows the 80% predictive interval.

### Predictive power of model

We characterized how forecast skill changed with the number of training years (Figure 6). Skill improved steeply over the first few training years and then plateaued. A segmented regression placed the breakpoint at approximately 4.3 years in spring (95% bootstrap CI 3.5–6.5) and 3.2 years in fall (95% bootstrap CI 2.5–4.5). Beyond these points, additional years of banding data yielded negligible improvement in forecast skill for either season (Figure 6).

**FIGURE 6:**
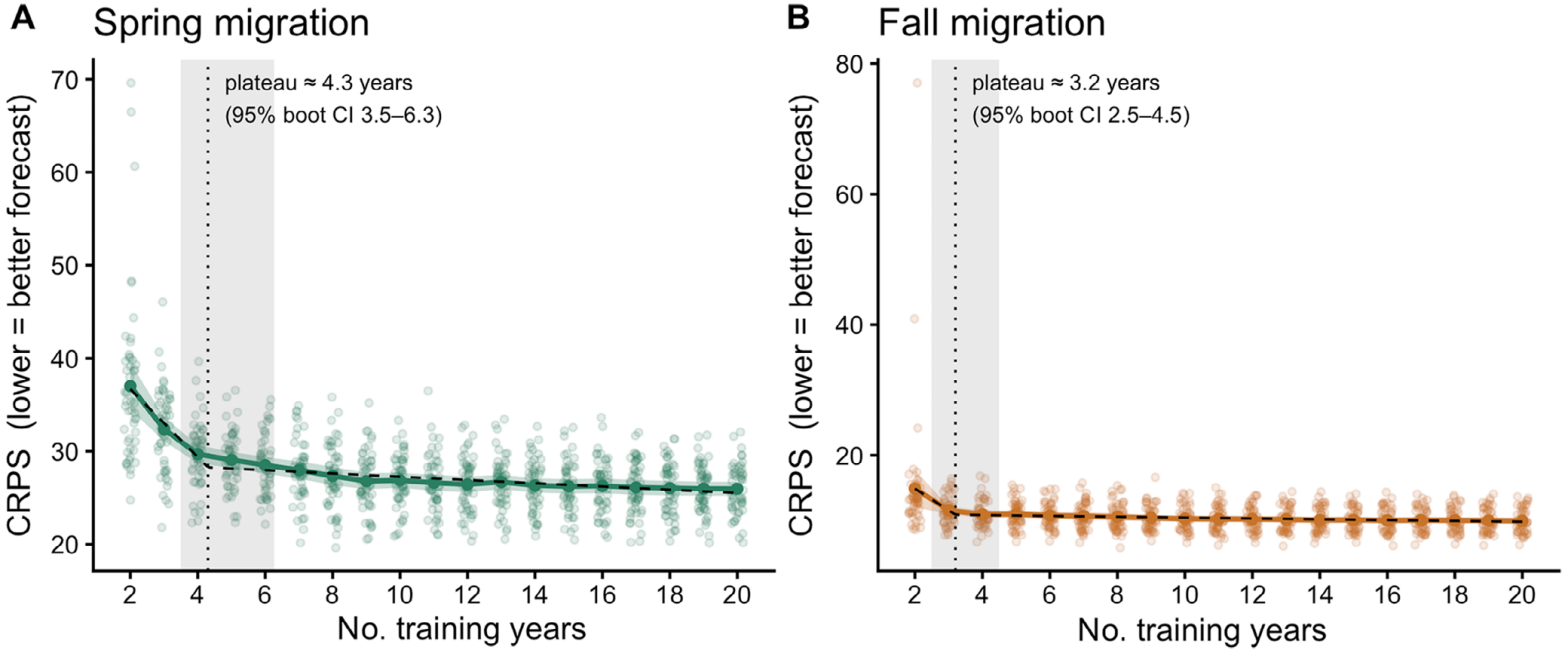
Forecast performance versus training‐set length, by season. For each training‐set size (2–20 years), the model was trained on a random subset of years and evaluated on 3 years of a randomly drawn fixed held‐out set. Points show the continuous ranked probability score (CRPS; lower = better forecast) for each of 50 replicates, and the solid line is the replicate mean (± 95% CI). The dashed line is a segmented (piecewise‐linear) regression fit and the vertical dotted line marks the estimated breakpoint, which denotes the training‐set size beyond which additional years yield negligible improvement. The shaded band shows a 95% bootstrap CI for the estimated breakpoint. For this analysis, CRPS was computed in closed form from each forecast's negative binomial predictive distribution (fitted mean and dispersion), rather than by posterior‐predictive simulation as elsewhere.

## DISCUSSION

The lower atmosphere serves as a conduit for the movement of thousands of species across the globe (Bauer et al., 2018), yet a full‐cycle understanding of bird migration requires connecting these aerial dynamics to terrestrial outcomes. We bridged this gap by combining WSR with bird banding records to predict when birds were captured on the ground, revealing a strong relationship between nightly migration detected by WSR and stopover events recorded at the Appledore Island Banding Station. Although our analysis focused on the broad assemblage of nocturnally migrating songbird species (~86% of species included and ~97% by abundance), with some notable exceptions (e.g., diurnally migrating Cedar Waxwings), we were able to apply the same framework scaled down to the species level. We predicted the 10 most common species captured at the banding site during both spring and fall, demonstrating that WSR data can guide species‐level ecological insights to inform conservation decisions. This approach has broad implications, enabling forecasts not only of birds in the airspace (Bradarić et al., 2024; Lippert et al., 2024), but also on the ground.

Our forecasts strengthened the linkage between aerial migration intensity and terrestrial capture rates in both seasons. However, environmental and radar predictors added more forecast skill—relative to a persistence baseline—in spring than in fall (median CRPS skill 0.25 vs. 0.12), indicating that a larger share of fall capture dynamics is governed by short‐term processes not captured by our aerial and atmospheric predictors. This weaker environmental signal in fall likely reflects increased behavioral and environmental complexity (Horton, Van Doren, Stepanian, Farnsworth, & Kelly, 2016; Nilsson et al., 2013), as migrants often delay movement and accumulate under unfavorable conditions before resuming migration during brief windows of favorable weather conditions (Richardson, 1978). As a result, stopover dynamics in fall may integrate atmospheric conditions across multiple nights rather than responding primarily to conditions immediately preceding banding events. Longer stopover durations documented during fall migration further decouple nightly aerial movement from local capture rates (Morris et al., 1994). Consistent with this interpretation, previous‐day captures were by far the most influential predictor in the fall model (Appendix S1: Figure S2). Because we counted only newly banded birds, this day‐to‐day persistence likely reflects the gradual capture of individuals present over multiple days, and birds not caught on their first day may be banded on subsequent days. Additionally, fall banding operations at Appledore Island span a shorter portion of the migratory season, likely missing the late pulses of movement and further limiting model performance. Radar‐based analyses indicate that approximately 50% of migratory passage through Portland, Maine occurs between September 17th and October 13th (Horton et al., 2021), whereas fall banding operations at Appledore Island extend only from mid‐August through September 20th. As a result, our sampling window likely misses a substantial and ecologically important phase of autumn migration, which may further contribute to reduced model performance in fall. Integrating complementary data streams, such as individual‐level tracking from Motus radio telemetry (Taylor et al., 2017), would allow explicit quantification of stopover duration and movement decisions, providing a more mechanistic basis for linking aerial migration to terrestrial observations and improving fall forecasts.

The forecasting framework we present has the potential to extend well beyond avian songbird migration and may be able to predict other large‐scale movement events. Many different aerial movements can be captured by WSR, although broad front migration has dominated the literature in the last decade (Hüppop et al., 2019; Nilsson et al., 2018; Shi et al., 2023; Sivakumar et al., 2021; Weisshaupt et al., 2021). These movements may include nocturnal and diurnal insect migrations (e.g., Painted Lady, Monarch, Moth, Grasshopper) (Klein et al., 2021; Menz et al., 2022; Talavera & Vila, 2017; Tielens et al., 2022), diurnal migrations of soaring birds (e.g., hawks, falcons, vultures, pelicans, cranes) (Shannon et al., 2002), waterfowl movements (Williams & Laird, 2018), and nightly bat movements (Stepanian & Wainwright, 2018). Strong linkages between migratory movements and atmospheric conditions have been well documented in many of these case examples (Cormont et al., 2011; Drake & Farrow, 1988; Smith & McWilliams, 2016; Vansteelant et al., 2014), however, a primary limitation remains the availability of broad‐scale training data from both ground observations and annotated radar imagery. Participatory science records (e.g., iNaturalist, eBird) may be obvious points of integration to broaden the footprint of ecological forecasts (Callaghan et al., 2021). The ability to forecast movements across diverse taxa has broad applications, including disease ecology (Peterson et al., 2009), aviation safety (van Gasteren et al., 2019), and aerial‐structure collision avoidance (Bauer et al., 2017).

We selected the Appledore Island Migration Station for developing our forecasts due to its geographic isolation and extensive, multi‐decade banding records, which provided a robust training dataset. A key question, however, is whether model performance depends on the length of the training timeseries—specifically, how many years of data are necessary to generate a reliable forecast? The long‐term dataset from Appledore allowed us to simulate different scenarios, revealing that at least 3–6 years of banding data were needed for performance stabilization. This suggests that even shorter term banding datasets can be effectively leveraged, expanding the applicability of our forecasting model. For example, Neate‐Clegg and Tingley identified 97 North American banding sites with at least 20 years of spring migration data and 271 sites with comparable fall data (Neate‐Clegg & Tingley, 2023). This significantly broadens the potential for implementing our approach, both within North America (Horton, Morris, et al., 2023) and globally, as demonstrated by similar long‐term banding efforts methodology (Jiao et al., 2016; Petras & Vrezec, 2022; Ruiz‐Gutiérrez et al., 2012).

While our predictive models successfully linked birds detected by WSR in the airspace to those banded on the ground, it remains unclear whether this pattern is site‐specific or taxa‐specific. Given Appledore's geographic isolation—particularly concerning songbird stopover habitat—we hypothesized that migrants' selection criteria for stopover would be relatively simplified. Mechanistically, stopover inputs to the island should be strongly influenced by nocturnal migration activity, a pattern we observed in both spring and fall (Appendix S1: Figures S1 and S2). However, even within the Isle of Shoals, stopover selection varies, with some migrant species preferentially using Appledore Island while rarely stopping at nearby Star Island (Suomala et al., 2012). Expanding this analysis to additional banding locations will help determine whether our model's predictive capacity is geographically constrained to coastal and island settings or if it extends to inland banding operations (e.g., Powdermill Avian Research Center). These results suggest that isolated patches of suitable habitat may function as ecological “islands” during migration, embedded within broader matrices of unsuitable habitat and encountered by migrants over short temporal scales. In the Midwestern United States, for example, extensive agricultural landscapes may create ecological boundaries for migratory birds similar to those encountered before crossing large bodies of water (Guo et al., 2023), leading to the formation of isolated stopover regions akin to islands. As anthropogenic habitat fragmentation increases, these isolated stopover sites may become a defining feature of the broader migratory landscape (Horton, Buler, et al., 2023).

Ecological forecasting involves predicting ecosystem dynamics while accounting for uncertainties and explicitly considering environmental conditions (Clark et al., 2001). In this study, we developed an ecological forecast to predict the number of migratory birds captured at a coastal banding site, using atmospheric conditions and biological indices derived from WSR. By incorporating accurate atmospheric forecasts and nightly bird migration forecasts, both of which are currently generated with a 16‐day lead time (Global Forecast System (GFS), 2020; Van Doren & Horton, 2018), our approach can be extended from now‐casting (hours in advance) to near‐term ecological forecasting (days to weeks in advance), enabling actionable predictions that can inform conservation decisions, such as optimizing disease spread policies or planning field monitoring efforts in advance. Similar forecasting approaches could be used to predict the movements of insects (Chapman et al., 2008; Stepanian et al., 2020), bats (Horn & Kunz, 2008), and other migratory organisms, as well as ecological processes such as seed and spore dispersal (Stephen et al., 1990), pest outbreaks (Anjita et al., 2024), and disease spread (Lisovski et al., 2018). By linking atmospheric forecasts and remote sensing data to terrestrial or aquatic ecological responses, we can develop scalable and transferable forecasting models that bridge airspace, land, and water systems. Advancing these efforts will support timely, evidence‐based decision‐making in conservation, resource management, and public health. The tools and infrastructure to operationalize these forecasts now exist, and expanding these applications offers significant potential to proactively manage rapidly changing ecological dynamics across ecosystems.

## AUTHOR CONTRIBUTIONS

All authors contributed to writing and editing. Burt and Horton led the first draft of this manuscript. Horton led analysis and generated figures. Morris contributed bird banding data.

## CONFLICT OF INTEREST STATEMENT

The authors declare no conflicts of interest.

## Supporting information


Appendix S1.

## Data Availability

Data and code (Horton, 2026a, 2026b) are available in Figshare at https://doi.org/10.6084/m9.figshare.31161232.v1 and https://doi.org/10.6084/m9.figshare.31161280.v3.
