# Supplementary material for: What goes up must come down: Using weather surveillance radar data to forecast migratory birds banded on the ground
Source: Ecology. 2026 Nov 5;107(11):e70565. doi: 10.1002/ecy.70565 (PMC13740661; doi:10.1002/ecy.70565)
Supplement: Supplementary file 1 [file ECY-107-e70565-s001.pdf]

Appendix S1

What goes up must come down: Using weather surveillance radar data to forecast migratory birds banded on the ground

Carolyn S. Burt, Sara R. Morris, Siddharth Chidambaram, and Kyle G. Horton

Ecology

Expansion on the cross-validation analysis:

Our cross-validation is a leave-one-year-out approach, meaning that we withhold an entire migration year, trained on the remaining years, and predicted the held-out year. Withholding whole years means that we never interpolate within an autocorrelated daily sequence using its immediate neighbors. We view this approach as a very close approximation to how we would implement these models to make real world forecasts of banding events. However, to additionally confirm that our conclusions are not an artifact of the leave-one-year-out scheme, which can train on years later than the one being predicted, we additionally evaluated the models under an expanding-window (end-sample holdout) forecast, in which each year was predicted using only the years preceding it, a true out-of-sample forecast. Under this scheme the full model retained positive CRPS skill relative to the baseline in nearly all forecast years (spring: 17 of 18; fall: 19 of 21), confirming that our conclusions are robust to the validation approach. Median skill was essentially unchanged in fall (0.12 vs. 0.12 under leave-one-year-out) and remained substantial in spring, though somewhat lower (0.16 vs. 0.25). This seasonal difference is itself consistent with our training-length analysis because spring skill continues to improve gradually with additional training years while fall skill plateaus after only a few, the expanding window's smaller average training set lowers spring skill but leaves fall skill unaffected. We retain leave-one-year-out as our primary analysis because it maximizes the data length, with 23–26 years. We further note that our models contain no year term and therefore cannot exploit long-term trends, limiting the scope for information leakage between folds.

Table S1: Smooth-term summary for the final spring full GAM: estimated degrees of freedom (edf), reference degrees of freedom, χ^2, and approximate p-value for each term. Overall fit statistics (deviance explained, adjusted R^2, negative binomial θ, sample size n, and intercept) are given in the table footer.

Spring full model

Term	edf	Ref. df	Chi-sq	p-value
Previous-day captures	1.45	2	11.93	<0.001
KBOX reflectivity	1.76	2	24.75	<0.001
KGYX reflectivity	0.47	2	0.91	0.155
KGYX zonal ground speed	1.58	2	7.75	0.005
KBOX zonal ground speed	0.96	2	22.40	<0.001
KGYX meridional ground speed	1.75	2	18.13	<0.001
KBOX meridional ground speed	0.29	2	0.38	0.233
Visibility	0.00	2	0.00	0.765
Zonal wind speed (900 hPa)	1.90	2	56.70	<0.001
Meridional wind speed (900 hPa)	1.69	2	17.81	<0.001
Air temperature (900 hPa)	1.88	2	37.51	<0.001
Net hours	0.93	2	12.76	<0.001
Ordinal date	1.94	2	80.60	<0.001
Deviance explained = 57.8%; adj. R^2 = 0.47; θ = 3.27; n = 481; intercept = 4.177 (SE 0.026).				

Table S2. Predictor importance in the final spring and fall generalized additive models, quantified by leave-one-variable-out ΔAIC (the change in AIC when a term is removed; ΔAIC = AIC_reduced − AIC_full, with larger values indicating greater importance). For each predictor, the rank (1 = most important) and ΔAIC are given for both seasons; rows are ordered by spring rank. Atmospheric variables were extracted above Appledore Island at 900 hPa.

Predictor	Spring		Fall	
	Rank	ΔAIC	Rank	ΔAIC
Ordinal date	1	62.72	4	9.08
Zonal (W-E) wind speed	2	49.29	8	4.39
Air temperature	3	26.40	13	-0.14
KBOX reflectivity	4	17.01	6	6.43
Meridional (S-N) wind speed	5	12.63	3	9.79
KGYX meridional (S-N) ground speed	6	11.37	7	5.15
Net hours	7	10.83	2	19.74
Previous-day captures	8	9.23	1	158.66
KBOX zonal (W-E) ground speed	9	7.41	12	0.00
KGYX zonal (W-E) ground speed	10	4.84	9	1.88
Visibility	11	0.01	11	0.01
KBOX meridional (S-N) ground speed	12	-0.20	10	0.12
KGYX reflectivity	13	-0.50	5	8.96

Table S3: Smooth-term summary for the final fall full GAM: estimated degrees of freedom (edf), reference degrees of freedom, χ^2, and approximate p-value for each term. Overall fit statistics (deviance explained, adjusted R^2, negative binomial θ, sample size n, and intercept) are given in the table footer.

Fall full model

Term	edf	Ref. df	Chi-sq	p-value
Previous-day captures	1.95	2	184.89	<0.001
KBOX reflectivity	1.75	2	15.63	<0.001
KGYX reflectivity	1.82	2	16.90	<0.001
KGYX zonal ground speed	0.75	2	2.99	0.039
KBOX zonal ground speed	0.00	2	0.00	0.831
KGYX meridional ground speed	0.90	2	9.43	<0.001
KBOX meridional ground speed	1.18	2	3.23	0.060
Visibility	0.00	2	0.00	0.886
Zonal wind speed (900 hPa)	0.89	2	7.78	0.003
Meridional wind speed (900 hPa)	1.81	2	14.21	<0.001
Air temperature (900 hPa)	0.32	2	0.49	0.211
Net hours	0.96	2	23.64	<0.001
Ordinal date	1.40	2	13.03	<0.001

Deviance explained = 49.9%; adj. R^2 = 0.44; θ = 5.66; n = 689; intercept = 3.477 (SE 0.017).

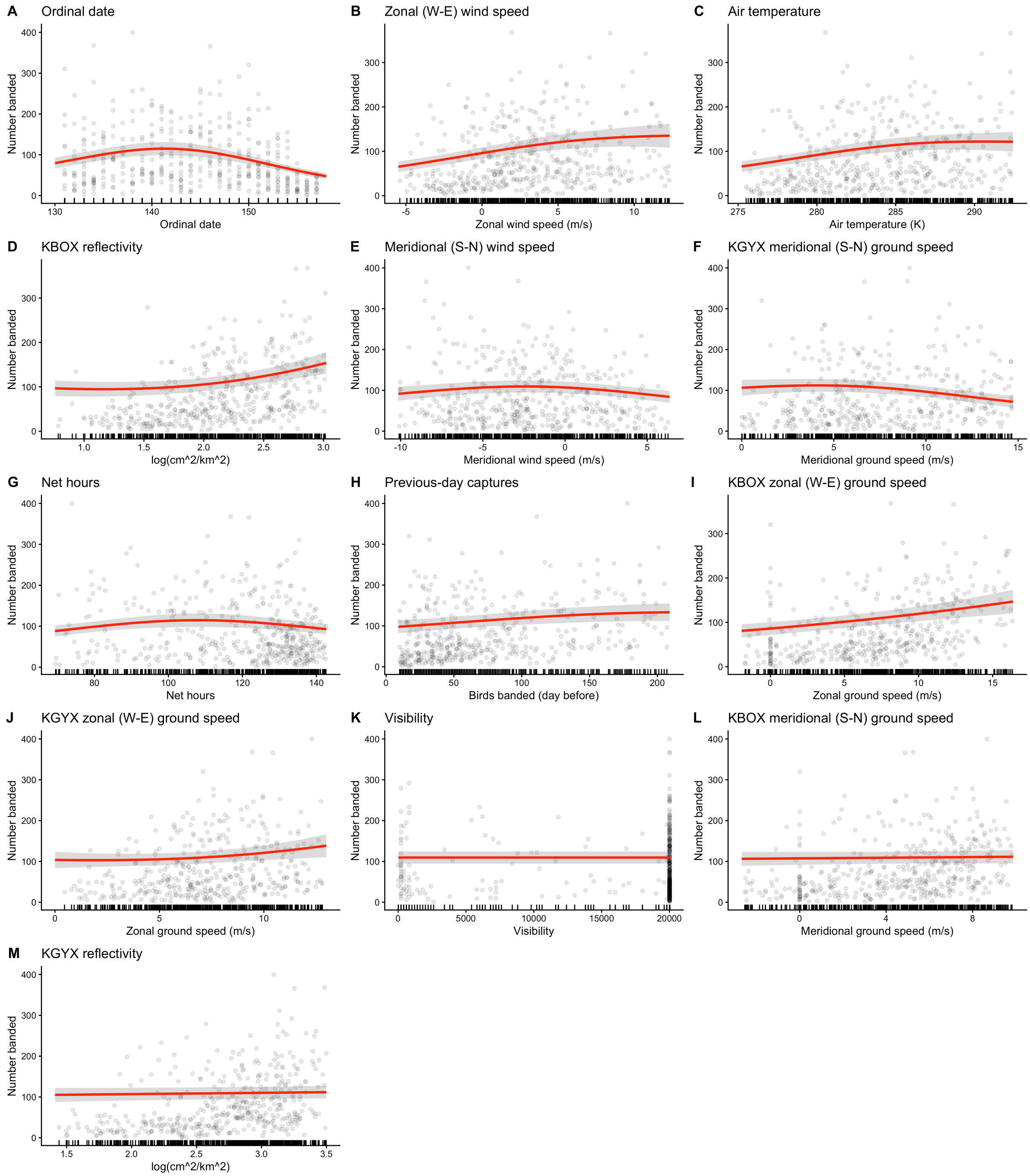

Figure S1. Partial-dependence plots for each predictor in the final spring generalized additive model, with panels ordered by decreasing variable importance (leave-one-variable-out ΔAIC; panel A most important). In each panel, the red line shows the predicted daily number of birds banded across the predictor's 5th–95th percentile range, holding all other predictors at their median values (net-hours fixed at 123); the shaded band is the approximate 95% confidence interval (± 1.96 SE). Grey points are the observed daily counts over the same range, and the rug marks the distribution of the predictor. Predictions are on the response scale.

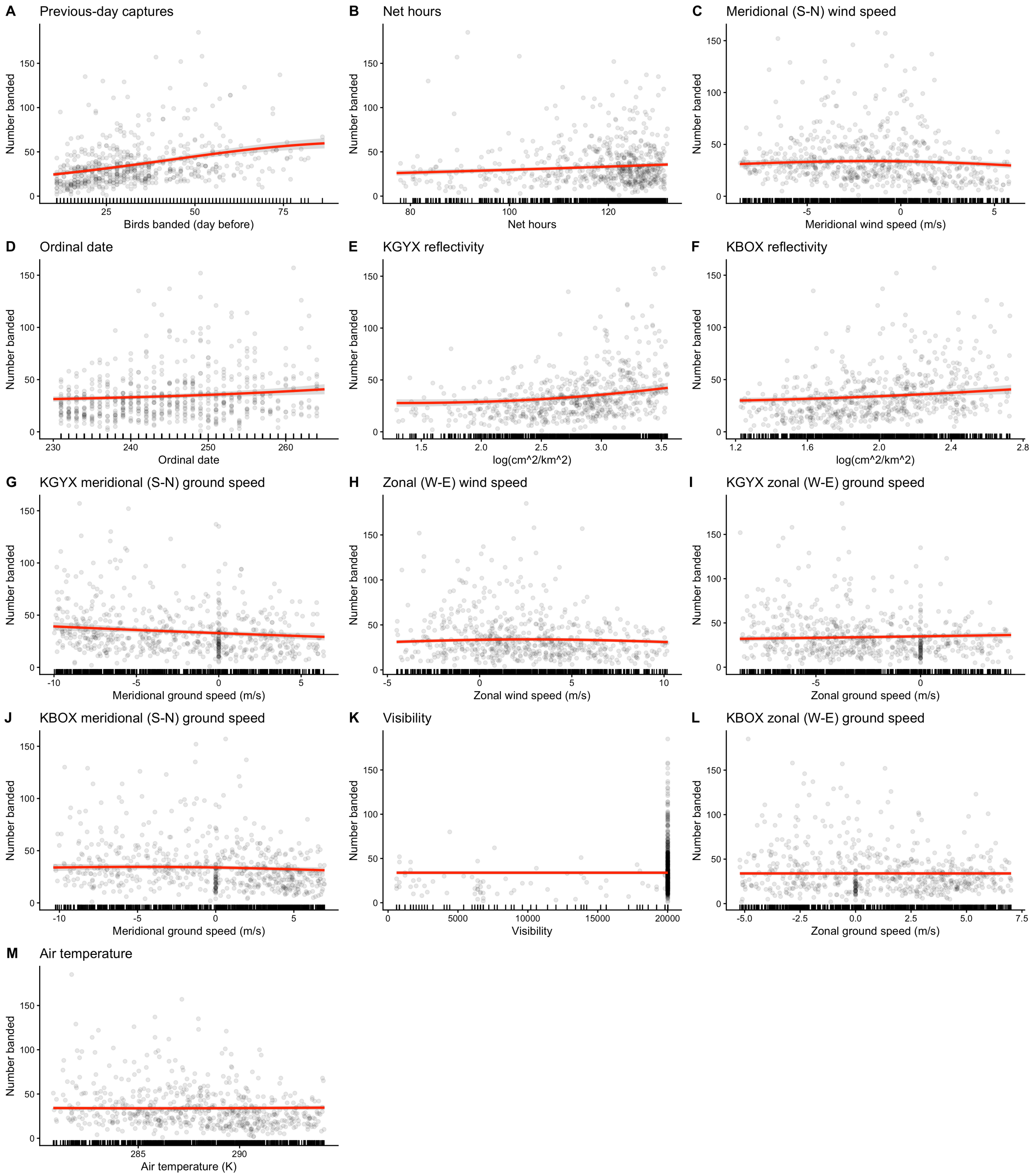

Figure S2. Partial-dependence plots for each predictor in the final fall generalized additive model, with panels ordered by decreasing variable importance (leave-one-variable-out ΔAIC; panel A most important). In each panel, the red line shows the predicted daily number of birds banded across the predictor's 5th–95th percentile range, holding all other predictors at their median values (net-hours fixed at 123); the shaded band is the approximate 95% confidence interval (± 1.96 SE). Grey points are the observed daily counts over the same range, and the rug marks the distribution of the predictor. Predictions are on the response scale.
